# CYCD3 D-type cyclins regulate cambial cell proliferation and secondary growth in *Arabidopsis*

**DOI:** 10.1093/jxb/erv218

**Published:** 2015-05-28

**Authors:** Carl Collins, N. M. Maruthi, Courtney E. Jahn

**Affiliations:** ^1^Natural Resources Institute, University of Greenwich, Central Avenue, Chatham Maritime, Kent ME4 4TB, UK; ^2^Department of Bioagricultural Sciences and Pest Management, Colorado State University, 1177 Campus Delivery, Fort Collins, CO 80523-1177, USA

**Keywords:** *Arabidopsis*, cambium, cell cycle, cell division, cell expansion, cyclin D3, inflorescence stem, organ size, secondary growth, vascular development, xylem.

## Abstract

A major proportion of plant biomass is derived from the activity of the cambium, a lateral meristem responsible for vascular tissue formation and radial organ enlargement in a process termed secondary growth. In contrast to our relatively good understanding of the regulation of primary meristems, remarkably little is known concerning the mechanisms controlling secondary growth, particularly how cambial cell divisions are regulated and integrated with vascular differentiation. A genetic loss-of-function approach was used here to reveal a rate-limiting role for the *Arabidopsis CYCLIN D3* (*CYCD3*) subgroup of cell-cycle genes in the control of cambial cell proliferation and secondary growth, providing conclusive evidence of a direct link between the cell cycle and vascular development. It is shown that all three *CYCD3* genes are specifically expressed in the cambium throughout vascular development. Analysis of a triple loss-of-function *CYCD3* mutant revealed a requirement for *CYCD3* in promoting the cambial cell cycle since mutant stems and hypocotyls showed a marked reduction in diameter linked to reduced mitotic activity in the cambium. Conversely, loss of *CYCD3* provoked an increase in xylem cell size and the expression of differentiation markers, showing that *CYCD3* is required to restrain the differentiation of xylem precursor cells. Together, our data show that tight control of cambial cell division through developmental- and cell type-specific regulation of *CYCD3* is required for normal vascular development, constituting part of a novel mechanism controlling organ growth in higher plants.

## Highlight

*Arabidopsis CyclinD3* genes are revealed as central regulators of cambial cell proliferation and vascular development, which constitutes part of a novel mechanism controlling secondary growth and radial organ size.

## Introduction

Plant growth and development relies on the co-ordination of cell division and cell expansion, which occur in the stem-cell systems of the meristems (Gonzalez *et al.*, 2012). The lateral meristem of the procambium is established during primary growth from the apical meristems and is responsible for forming the vascular (conductive) tissues of the primary xylem and primary phloem. In stems and roots of dicotyledonous plants, the procambium, as well as a subset of specialized parenchyma cells (as in stems), gives rise to a further lateral meristem, the cambium, which is responsible for the production of secondary xylem and secondary phloem, leading to the radial enlargement of the plant body in a process termed secondary growth. Vascular tissue formed through secondary growth constitutes the major proportion of plant biomass and is the basis of wood production in tree species. Vascular development in the *Arabidopsis* inflorescence stem is an attractive model system to study the co-ordination of cell division and cell expansion in organ growth as the two processes are spatially separated in radially expanding stems (Sehr *et al.*, 2010; Sanchez *et al.*, 2012). At the stem apex, the first of two specialized cambial cell populations, termed the fascicular cambium (FC), proliferates to form daughter cells that are displaced either inwards or outwards following a pathway towards terminal differentiation to adopt a xylem or phloem cell fate, respectively. Differentiation of cambial daughter cells is associated with cell expansion, lignification, and secondary cell wall biosynthesis (Schuetz *et al*., 2013). At the base of elongated stems, a second specialized cambial cell population termed the interfascicular cambium (IC) is established *de novo* from cells lying within interfascicular regions, culminating in a ring-like continuous meristematic zone constituting the vascular cambium (Sanchez *et al.*, 2012). Once formed, the vascular cambium undergoes rapid proliferation marking the major phase of secondary growth and radial organ expansion.

Tight control of the rate of cambial cell division and the timing of cell expansion/differentiation in cambial daughter cells is critical for the maintenance and size of the cambium and, ultimately, for determining radial organ size. These processes are known to be under developmental, environmental, and hormonal control (Miyashima *et al.*, 2013). However, as only a small number of genes have been identified as having roles in the control of cambial development and activity, remarkably little is known concerning the spatiotemporal regulation of cambial cell division and its integration with vascular differentiation.

Plant cell division is regulated by cyclin-dependent kinases (CDK) which require the binding of a positive regulatory cyclin subunit for promoting the entry and progression of cells through the cell cycle (Gonzalez *et al.*, 2012). In *Arabidopsis*, at least 110 genes have so far been recognized as belonging to a small number of gene families comprising the ‘core’ cell cycle machinery, including CDKs and cyclins, that act at different phases of the cell cycle to control division and the transition to differentiation (Vandepoele *et al.*, 2002; Menges *et al.*, 2005; Van Leene *et al.*, 2010). One gene family, the D-type cyclins (*CYCD*), play a central role in promoting cell division through their activation of the CYCD-RETINOBLASTOMA-RELATED (RBR)-E2F pathway at the G1-S checkpoint in response to mitogenic signals such as auxin and cytokinin (Riou-Khamlichi *et al.*, 1999; Oakenfull *et al.*, 2002; Dewitte *et al.*, 2007). The *CYCD* family is conserved between all plant species so far characterized including *Arabidopsis*, rice, and poplar, with individual gene members belonging to one of six subgroups (Menges *et al.*, 2007). In *Arabidopsis*, the CYCD3 subgroup consists of three members (CYCD3;1, CYCD3;2, and CYCD3;3) which were shown to control the balance between cell division and cell expansion in shoot lateral organs by regulating the duration of mitotic activity, as well as mediating the response of meristematic cells to cytokinin (Dewitte *et al.*, 2007). A partial expression analysis of *CYCD3;1* using *in situ* hybridization revealed activity in the procambium of developing leaves and inflorescence stems (Dewitte *et al.*, 2003). *CYCD* genes are thus prime candidates for playing roles in the regulation of cambial cell division and its integration with vascular differentiation.

The wider role of the cell cycle in vascular development and secondary growth has been investigated here. Based on public gene expression profiling data, it is revealed that a small set of core cell-cycle regulators are associated with both primary and secondary vascular development. The *CYCD3* subgroup is identified as positive regulators of cambial cell proliferation and secondary growth based on a detailed gene expression and loss-of-function analysis of the *CYCD3* genes during vascular development. Furthermore, an additional role for the *CYCD3* subgroup in restraining cell expansion and differentiation of xylem precursor cells is uncovered. Thus, tight control of the cambial cell cycle and co-ordination with cell expansion and differentiation processes through developmental- and cell-type-specific regulation of *CYCD3* is required for proper vascular development and radial organ growth.

## Materials and methods

### Plant material and growth conditions

*Arabidopsis thaliana* ecotype Columbia was used as the wild type in all experiments. The *pCYCD3;1:GUS*, *pCYCD3;2:GUS*, *pCYCD3;3:GUS*, and *cycd3;1–3* lines were as described by Dewitte *et al.* (2007) and were obtained from James Murray (Cardiff, UK). The genotype of the *cycd3;1–3* line was confirmed by the absence of transcripts for all three *CYCD3* genes using RT-PCR (see Supplementary Fig. S1 at *JXB* online). Seeds were surface-sterilized with 70% ethanol (v/v) for 3min, followed by 20% hypochlorite (v/v) for 15min, and rinsed six times with sterile deionized water. Sterilized seed were sown onto square Petri plates containing sterile, solid, half-strength Murashige and Skoog (MS) medium. Plates were shifted to a cold room at 4 °C for 3 d to synchronize germination, then transferred to a growth chamber and grown at 22±2 °C under a 16/8h light/dark photoperiod. For the analysis of mature plants, 11-d-old seedlings were transplanted from the growth medium to soil (Fafard 4P Mix; Conrad Fafard, USA) contained in rectangular plastic tray inserts (6×3 compartments) inside black trays with humidity domes. Each seedling was placed in a separate compartment in each insert (to ensure uninhibited growth to maximum height). Trays were moved to a growth chamber and grown at 22±2 °C under a 16/8h light/dark photoperiod at 60% humidity. Humidity domes were removed after 3 d growth.

### Conventional reverse transcription-PCR and reverse transcription quantitative real-time PCR

Tissue was obtained from the top (5mm under the shoot meristem), middle, and at the base (immediately above the rosette) of inflorescence stems from 30-cm-tall plants. Dissected tissue was collected from five individual plants for each stem position and pooled for RNA isolation. Total RNA was extracted using TRIzol Reagent (Invitrogen) and further purified using the DNA-free Kit (Ambion) according to the manufacturer’s instructions. RNA concentration and purity were determined using a NanoDrop 2000 Spectrophotometer (Thermo Scientific). RNA integrity was visualized by denaturing agarose gel electrophoresis and ethidium bromide staining. All reverse transcriptions were performed using 2 μg total RNA in a total reaction volume of 20 μl with the RETROscript kit (Ambion) using an oligo (dT)_18_ primer according to the manufacturer’s instructions. Briefly, samples were heated to 70 °C for 3min, held at 42 °C for 1h in the presence of 1 μl MMLV-RT, and inactivated at 92 °C for 10min. After cDNA synthesis, all samples were diluted 10 times in molecular biology grade water (Thermo Scientific) and stored at –20 °C.

All standard PCR reactions were performed using *Taq* DNA Polymerase (New England BioLabs) according to the manufacturer’s instructions. All quantitative real-time PCR (qPCR) reactions were prepared in twin.tec 96-well PCR plates (Eppendorf). Each 25 μl reaction consisted of 12.5 μl 2× QuantiTect SYBR Green PCR Master Mix (Qiagen), 1.5 μl forward and reverse primer (final concentration of 0.3 μM each primer), 3.0 μl diluted cDNA, and 8.0 μl molecular biology grade water (Thermo Scientific), typically prepared using master-mixes. Triplicate reactions from two biological replicates were performed on a Mastercycler ep *realplex* cycler (Eppendorf) using the following thermal cycling profile: 95 °C for 15min (to activate the polymerase), followed by 45 cycles of 94 °C for 15 s, 60 °C for 30 s, and 72 °C for 30 s. Finally, a dissociation (melting curve) analysis was performed to confirm the amplification of a single expected PCR product and the absence of primer dimer formation, according to the manufacturer’s instructions (Eppendorf). Minus reverse transcription and no template control reactions were included in all assays to confirm the absence of contaminating DNA in RNA preparations and PCR reagents, respectively. Normalized relative expression levels were calculated using the ΔΔCt method (Pfaffl, 2001) using the *ACTIN2* gene as the endogenous control (Collins *et al.*, 2012). Primers were designed using nucleotide sequences from The *Arabidopsis* Information Resource (TAIR; http://www.arabidopsis.org). The specificity of all primer sets for their respective target sequence was demonstrated by: (i) amplification of a single PCR product followed by sequencing of the resulting PCR fragment; (ii) generation of a single peak in the melting curve analysis; (iii) an *in silico* specificity screen using BLAST analysis (NCBI). The following primer pairs were used: *ACTIN2*, 5′-GAAGAACTATGAATTACCCGATGGGC-3′ and 5′-CCCGGGTTAGAAACATTTTCTGTGAACG-3′ (Collins *et al.*, 2012); *CYCD3;1*, 5′-GCAAGTTGATCCCTTTGACC-3′ and 5′-CAGCTTGGACTGTTCAACGA-3′ (Collins *et al.*, 2012); *CYCB1;1*, 5′-TAAGCAGATTCAGTTCCGGTCAAC-3′ and 5′-GGGAGCTTTACGAAAGAAATACTCC-3′ (Collins, 2008); *IRX3*, 5′-GCGTGTGCACCCATATCC-3′ and 5′-TCATCC ATTCTTTCCCGCC-3′ (Etchells and Turner, 2010); *SND2*, 5′-CAGACTCAACCACGTCAATGC-3′ and 5′-AGGGATAAAA GGTTGAGAGTC-3′ (Zhong *et al.*, 2008).

### Histology and microscopy

Histochemical staining for GUS activity in seedling and adult tissues was performed as described by Jefferson *et al.* (1987). GUS-stained and unstained tissue was cleared in 70% ethanol and mounted in 10% glycerol prior to microscopic analysis. Stem samples for sectioning were taken from (i) the base of inflorescences immediately above the rosette, and (ii) from 5mm below the shoot apex, of 30-cm-long plants in which the first internode was at least 3.5cm long. Samples were fixed in FAA (formalin/acetic acid/alcohol) and then dehydrated and embedded in paraffin, and microtome-cut sections 5 μm thick were stained with 0.05% toluidine blue. Whole plants were examined and photographed using a Leica S6 D dissecting microscope equipped with a Nikon coolpix 4500 digital zoom camera. Sectioned material was examined and photographed using a Zeiss Axioskop microscope equipped with a Jenoptik ProgRes digital zoom camera and ProgRes CapturePro (2.8.8) camera control software (Jenoptik). Adobe Photoshop CS5 (v12.1 x64) and Adobe Illustrator CS5 (v15.1.0) were used for image preparation.

### Phenotypic analyses

In all quantitative measurements described, sampled plants were taken from a total population of 108 plants. Stem diameter was measured at the base of inflorescences immediately above the rosette when the inflorescence was 1, 10, 20, and 30cm long. Hypocotyl diameter was measured at the base of the hypocotyl adjacent to the rosette in 30-cm-tall plants. Stem lengths were measured at 21, 28, 35, 42, 49, 56, and 63 d from the date of sowing. In the analyses of stem and hypocotyl diameter, and stem length, ten individual randomly-selected plants were evaluated at each time point. Measurement of the width:length (tangential:radial) ratio of vascular bundles at the stem base was as described previously (Fisher and Turner, 2007), by evaluating three randomly selected vascular bundles each from tissue sections from 15 individual randomly selected plants. Measurement of the lateral extension of the FC-derived (FCD) and IC-derived (ICD) tissues at the stem base was as described earlier (Sehr *et al.*, 2010), by evaluating both FCD and ICD extension at five randomly selected positions each from tissue sections from 15 individual randomly-selected plants. Cell numbers at the stem base were calculated from tissue sections from 15 individual randomly selected plants. Xylem cell sizes at the stem base were measured using ImageJ software v1.46r (http://rsbweb.nih.gov/ij/) from tissue sections from ten individual randomly selected plants. Briefly, images were adjusted (brightness/contrast) to reduce background noise using Adobe Photoshop CS5 (v12.1 x64). In ImageJ, images were converted to greyscale, and then to binary (threshold), followed by manual adjustments to remove further background noise. Cells were highlighted using the Outlines tool, and the Analyze Particles tool was used to obtain cell sizes (Calculated from cell area in μm^2^). Data were exported as a spreadsheet for further analysis. All statistical analyses were performed using JMP v10.0.2 software package (SAS Institute). A two-tailed independent Student’s *t* test was applied to data using significance levels of *P* <0.05, *P* <0.01, and *P* <0.001 as indicated by single, double, and triple asterisks, respectively.

### Extraction and analysis of data from gene expression databases

The *Arabidopsis* Gene Expression Database (AREX Lite; Brady *et al.*, 2007; http://www.arexdb.org/download.html) was used to extract expression levels of core cell-cycle and cambium marker genes in different cells and tissues of the root tip (Brady *et al.*, 2007). In this dataset, a mixed-model analysis was used to normalize all arrays globally (Brady *et al.*, 2007). Briefly, expression values were mean-normalized (centring to zero) and log_2_-transformed to yield relative expression indices. Therefore, high expression and low expression for any given gene (probe) is relative to zero (lowest expression). Supplementary Table S1 from Zhao *et al.* (2005) was the source of data for expression levels of core cell-cycle genes in phloem/cambium, xylem, and non-vascular tissues of the mature hypocotyl. Hierarchical clustering was performed using Cluster software v3.0 (Eisen *et al.*, 1998; De Hoon *et al.*, 2004; http://bonsai.hgc.jp/~mdehoon/software/cluster/) and visualized using Java TreeView v1.1.6r4 (Saldanha, 2004; http://jtreeview.sourceforge.net/).

## Results

### Global expression profiling of core cell-cycle genes identifies CYCD3 D-type cyclins as potential regulators of vascular development

To explore the wider role of the cell cycle in vascular development and to identify candidate cell-cycle genes that promote cambial cell proliferation, the transcriptional regulation of all known core cell-cycle genes was examined using data from high resolution *Arabidopsis* cell- and tissue-specific microarray profiles derived from the root tip (Brady *et al.*, 2007) and mature hypocotyl (Zhao *et al.*, 2005). The combination of both datasets essentially provides broad coverage of cambium-associated expression during both primary and secondary vascular development. In *Arabidopsis*, 110 core cell-cycle genes have been recognized (Vandepoele *et al.*, 2002; Menges *et al.*, 2005; Van Leene *et al.*, 2010) and, of these, 96 are represented by ATH1 array probes. Their transcription profiles weree first examined across 19 different cell and tissue types of the root tip (Brady *et al.*, 2007), and included data on four classical markers of cambial activity (*MP/ARF5*, *WOL*, *ATHB8*, and *PIN1*; Hardtke and Berleth, 1998; Mähönen *et al.*, 2000; Scarpella *et al.*, 2004, 2006) in order to correlate cell-cycle gene transcriptional activity with vascular stem cell division. This showed different core cell-cycle regulators to have distinct expression patterns in the root, with the majority of genes showing low overall expression variation across all cell and tissue types and thus low cell- and tissue-specificity (see Supplementary Fig. S2 at *JXB* online). As expected, the four cambium markers showed enrichment in cells and tissues derived from the vasculature confirming that the root expression map (Brady *et al.*, 2007) can serve as a reliable approximation of a vascular tissue-specific profile.

**Fig. 1.**
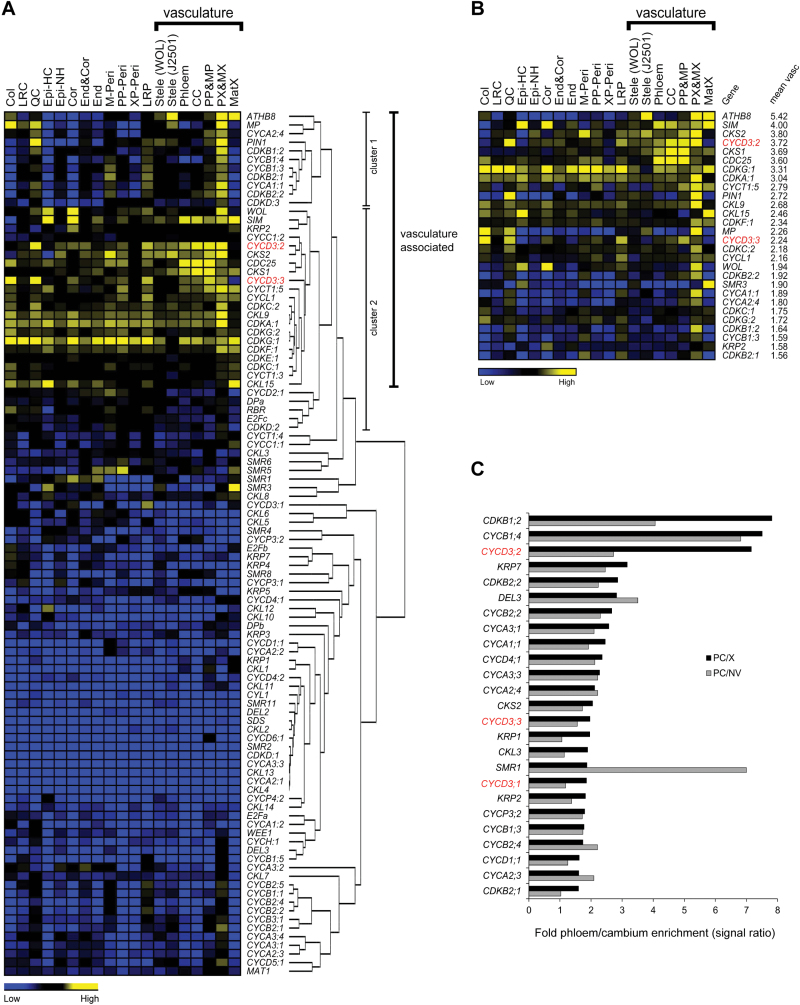
Expression patterns and clustering analysis of core cell-cycle genes in specific cell and tissue types of the root and mature hypocotyl. (A) Heat map representation of hierarchical clustering of 96 core cell-cycle genes and four cambium marker genes based on expression profile data across 19 different cell and tissue types of the root tip (Brady *et al.*, 2007). The solid bar indicates the seven cell and tissue types comprising the vasculature. High and low levels of expression are indicated by yellow and blue, respectively. The expression levels shown are relative to zero based on global normalization method (Brady *et al.*, 2007). Two distinct clusters of co-regulated genes showing broadly ‘vasculature-associated’ expression are indicated (cluster 1 and cluster 2). Cell and tissue types from left to right are columella (Col), lateral root cap (LRC), quiescent centre (QC), epidermis hair cell (Epi-HC), epidermis non-hair cell (Epi-NH), cortex (Cor), endodermis and cortex (End&Cor), endodermis (End), mature pericycle (M-Peri), phloem pole pericycle (PP-Peri), xylem pole pericycle (XP-Peri), lateral root primordia initials (LRP), stele using WOL marker [Stele (WOL)], stele using J2501 marker [Stele (J2501)], phloem and companion cell (Phloem), companion cell (CC), protophloem and metaphloem (PP&MP), protoxylem and metaxylem (PX&MX), mature xylem (MatX). (B) Ranking of 96 core cell-cycle genes and four cambium marker genes according to their average expression level across the seven cell and tissue types comprising the vasculature (indicated by the solid bar), based on expression profile data of the root tip (Brady *et al.*, 2007). Only the top 28 highest vasculature-expressed genes are shown and are displayed with their corresponding expression patterns across the root. Abbreviations used are as shown in (A). (C) Ranking of core cell-cycle genes according to their degree of phloem/cambium (PC) enrichment calculated as fold-differences between mean array signal intensities in PC versus xylem (X) tissue (PC/X), and in PC versus non-vascular (NV) tissues (PC/NV), using microarray data from the mature hypocotyl (Zhao *et al.*, 2005). Only the top 25 highest PC-enriched genes are shown.

Cluster analysis of core cell-cycle and cambium marker gene expression profiles showed that genes exhibiting a relatively constant, low expression level across the root tip generally cluster together (comprising the major proportion of genes) and revealed several separate clusters comprising co-regulated genes sharing vascular tissue-associated activity (Fig. 1A). Outside the main cluster of genes with constant low expression, two clusters of regulated genes can be broadly defined; the first cluster contains genes clearly enriched in the vasculature, quiescent centre (QC), and lateral root primordia (LRP), and including *ATHB8*, *MP*, and *PIN1*, and a second cluster containing genes generally enriched in the vasculature but showing more variable expression peaks in other cell layers and including *WOL* (Fig. 1A). The second cluster also contained a small subset of genes which exhibited a more constant high level of expression across all cell layers, exemplified by *CDKA;1* and *CDKG;1* (Fig. 1A). Having a broadly shared pattern of specificity for the vasculature, cluster 1 and cluster 2 were designated, with the exception of *CYCD2;1*, *DPa*, *RBR*, *E2Fc*, and *CDKD;2*, as ‘vasculature-associated’ comprising 28 cell-cycle genes and four cambium marker genes. All 96 core cell-cycle genes and four cambium marker genes were ranked according to their average expression levels across all seven cell and tissue types representing the vasculature (Fig. 1B). The most highly vasculature-enriched genes included all four cambium markers, accompanied by representative gene members from almost all the main core cell cycle gene families (Vandepoele *et al.*, 2002; Menges *et al.*, 2005; Van Leene *et al.*, 2010). Importantly, the vast majority of genes identified as ‘vasculature-associated’ (Fig. 1A), also had high overall expression in the vasculature (Fig. 1B), suggesting cell-cycle genes in this cluster have distinct roles in primary vascular development.

The expression patterns of core cell-cycle genes were examined next using data from transcriptional profiling of the mature hypocotyl (Zhao *et al.*, 2005). Calculations of fold-differences between mean array signal intensities in phloem/cambium (PC) versus xylem (X) tissue (PC/X), and in PC versus non-vascular (NV) tissues (PC/NV) (Zhao *et al.*, 2005), revealed a group of 25 cell-cycle genes showing high specificity for phloem/cambium tissue suggesting distinct roles in secondary vascular development (Fig. 1C). A significant overlap was noted between the set of 25 PC-specific cell-cycle genes in the mature hypocotyl (Fig. 1C), and the set of 28 cell-cycle genes identified as ‘vasculature-associated’ in the root tip (Fig. 1A). Indeed, 11 cell-cycle genes were shared between both sets. This suggests that a common set of core cell-cycle genes are involved in both primary and secondary vascular development.

As we were primarily interested in identifying core cell-cycle genes that are involved in promoting cambial cell divisions, the root and hypocotyl microarray profiles (Fig. 1) were scrutinized for positive cell-cycle regulators having a clear vascular tissue-associated profile. Close inspection of the data shows that the *CYCD3* class of D-type cyclins appear prominently among vasculature-enriched genes expressed during both primary and secondary vascular development (Fig. 1A–C; see Supplementary Fig. S2 at *JXB* online). The *CYCD3* subgroup has already been shown to be an important positive cell-cycle regulator of cell division in developing leaves and embryos (Dewitte *et al.*, 2007; Collins *et al.*, 2012). Consistent with a proposed role for CYCD3 in vascular development, a partial expression analysis of *CYCD3;1* using *in situ* hybridization revealed activity in the procambium of developing leaves and inflorescence stems (Dewitte *et al.*, 2003). Strikingly, it was found that the *CYCD3* genes also show peaks of activity in the QC and LRP, a pattern shared with known cambium regulators such as *PIN1* and *MP* (Fig. 1A; Hardtke and Berleth, 1998; Scarpella et al., 2004, 2006), providing further confirmation of a close association between *CYCD3* expression and vascular stem cell-associated activity. In light of these results, the decision was taken to focus our molecular investigations to elucidate the role of CYCD3 in vascular development.

### *CYCD3* genes are expressed in the cambium throughout development

**Fig. 2.**
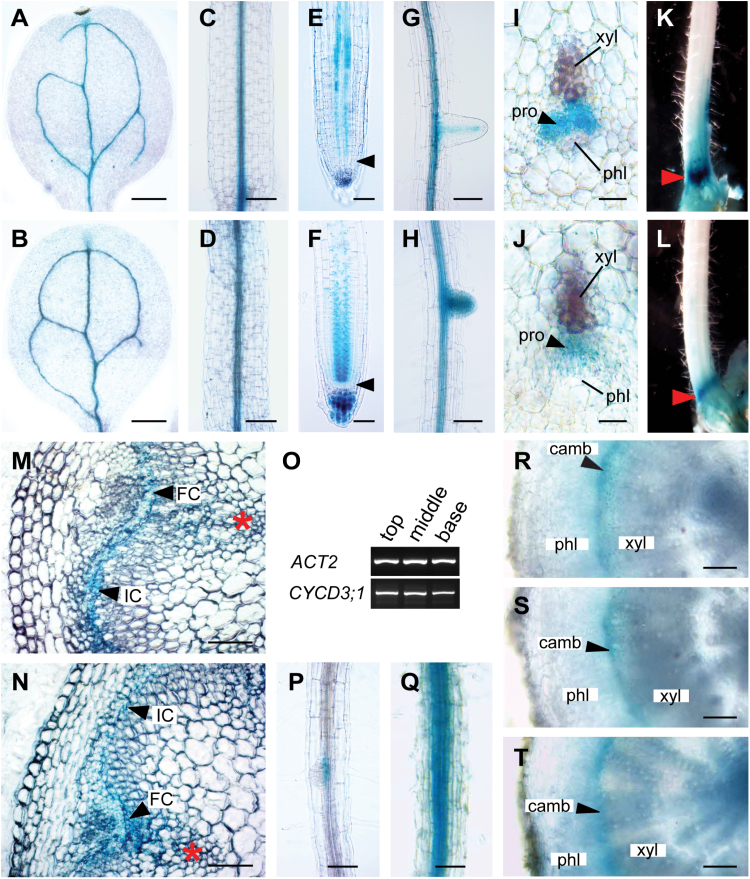
*CYCD3* genes are specifically expressed in the procambium and cambium during vascular development. (A–H) Images of 7-d-old seedlings showing *pCYCD3;2:GUS* (A, C, E, G) and *pCYCD3;3:GUS* (B, D, F, H) activity in the vasculature of the cotyledon (A, B), hypocotyl (C, D), root tip (E, F), and mature root zone and lateral root (G, H). In (E) and (F), the root apical meristem is indicated by arrowheads. (I, J) Transverse sections at 5mm below the shoot apex of 30-cm-long inflorescences showing *pCYCD3;2:GUS* (I) and *pCYCD3;3:GUS* (J) activity restricted to the procambium. (K, L) *pCYCD3;2:GUS* (K) and *pCYCD3;3:GUS* (L) staining accumulates at the base of 30-cm-long inflorescences (indicated by arrowheads) corresponding to the zone of maximum IC initiation and secondary growth. (M, N) Transverse sections at the base of 30-cm-long inflorescences immediately above the rosette reveal *pCYCD3;2:GUS* (M) and *pCYCD3;3:GUS* (N) activity restricted to fascicular cambium (FC) and interfascicular cambium (IC) (indicated by arrowheads). Asterisks indicate primary vascular bundles. (O) RT-PCR analysis reveal *CYCD3;1* transcripts at the top, middle, and base of 30-cm-long inflorescences (*ACT2* was used as endogenous control). (P, Q) *pCYCD3;1:GUS* expression was detected in root vascular tissue in 7-d-old seedlings (P), and in mature roots in 30-cm-tall plants (Q). (R-T) Transverse sections at the hypocotyl base from 30-cm-tall plants reveal *pCYCD3;1:GUS* (R), *pCYCD3;2:GUS* (S), and *pCYCD3;3:GUS* (T) expression restricted to the cambium. camb, cambium; FC, fascicular cambium; IC, interfascicular cambium; phl, phloem; pro, procambium; xyl, xylem. Scale bars=250 μm (A, B), 100 μm (C, D, G, H, M, N, P, Q, R, S, T), 50 μm (E, F), 25 μm (I, J).

To investigate a functional role for CYCD3 in vascular development, an attempt was made to determine more precisely the *in planta* expression of *CYCD3* genes using available GUS reporter transgenic plants. *CYCD3;1* has already been shown to be expressed in the procambium of developing leaves and inflorescence stems (Dewitte *et al.*, 2003). A preliminary expression analysis showed *CYCD3;2* and *CYCD3;3* to be expressed in seedling vascular tissue (Collins, 2008). However, this study did not achieve the spatial resolution required to determine whether these genes are specifically active in vascular stem cells, as found for *CYCD3;1* (Dewitte *et al.*, 2003). Therefore, we focused on determining the detailed tissue- and cell-specific expression of *CYCD3;2* and *CYCD3;3* during vascular tissue formation at different plant developmental stages using the GUS reporter lines *pCYCD3;2:GUS* and *pCYCD3;3:GUS*, respectively (Dewitte *et al.*, 2007). Both genes shared a remarkable degree of overlap in their expression domains with activity closely associated with vascular tissue initiation from seedling to adult plant stages (Fig. 2). In 7-d-old seedlings, *pCYCD3;2:GUS* and *pCYCD3;3:GUS* showed strong activity in developing vasculature of cotyledons, leaves, hypocotyls, and roots (Fig. 2A–H). In roots, *pCYCD3;2:GUS* and *pCYCD3;3:GUS* expression marked the two procambial cell files, beginning first in the root apical meristem (RAM) with persistent activity in the elongation and maturation zones (Fig. 2E, F), and throughout the mature root zones and in emerging lateral roots (Fig. 2G, H). Examination of transverse sections from 5mm below the shoot apex of adult stems, corresponding to the zone where vascular development is initiated, showed *pCYCD3;2:GUS* and *pCYCD3;3:GUS* expression specifically in mitotically active procambial cells (Fig. 2I, J). In intact whole 30-cm-long stems, an accumulation of *pCYCD3;2:GUS* and *pCYCD3;3:GUS* staining was observed in a narrow region at the stem base immediately above the rosette leaves (Fig. 2K, L), corresponding to the zone of maximum interfascicular cambium (IC) initiation and secondary growth identified previously (Sehr *et al.*, 2010). Transverse sections across this zone in both reporter lines confirmed GUS activity specifically in the fascicular cambium (FC) and IC (Fig. 2M, N).

Examination of the stem base from a GUS reporter line representing the *CYCD3;1* gene, *pCYCD3;1:GUS* (Riou-Khamlichi *et al.*, 1999), failed to show any staining (data not shown). However, consistent with previous expression studies of *CYCD3;1* in the stem (Dewitte *et al.*, 2003), *CYCD3;1* transcripts were detected at the base (and top and middle) of mature stems using RT-PCR (Fig. 2O), suggesting that the *pCYCD3;1:GUS* line may not entirely recapitulate the true expression pattern for *CYCD3;1* in the stem. Nevertheless, *pCYCD3;1:GUS* activity was observed in the root vasculature, which became progressively stronger as the roots reached mature stages, coinciding with the onset of root secondary growth (Fig. 2P, Q). Transverse sections of mature hypocotyls from 30-cm-tall plants revealed *pCYCD3;1:GUS*, *pCYCD3;2:GUS*, and *pCYCD3;3:GUS* expression specifically in the mitotically active cells of the vascular cambium, coinciding with the major phase of secondary growth in the hypocotyl (Fig. 2R–T). The expression patterns determined for all three *CYCD3s* using GUS reporters were in strong agreement with the results obtained from microarray profiling of the root tip and mature hypocotyl (Fig. 1). Together, the overlapping spatiotemporal patterns of *CYCD3* transcriptional regulation observed confirm a close association of *CYCD3* expression with active procambial/cambial cell proliferation throughout development, consistent with a distinct, subgroup-specific role for CYCD3 in vascular tissue formation.

### Loss of *CYCD3* causes reduced secondary growth and organ size

**Fig. 3.**
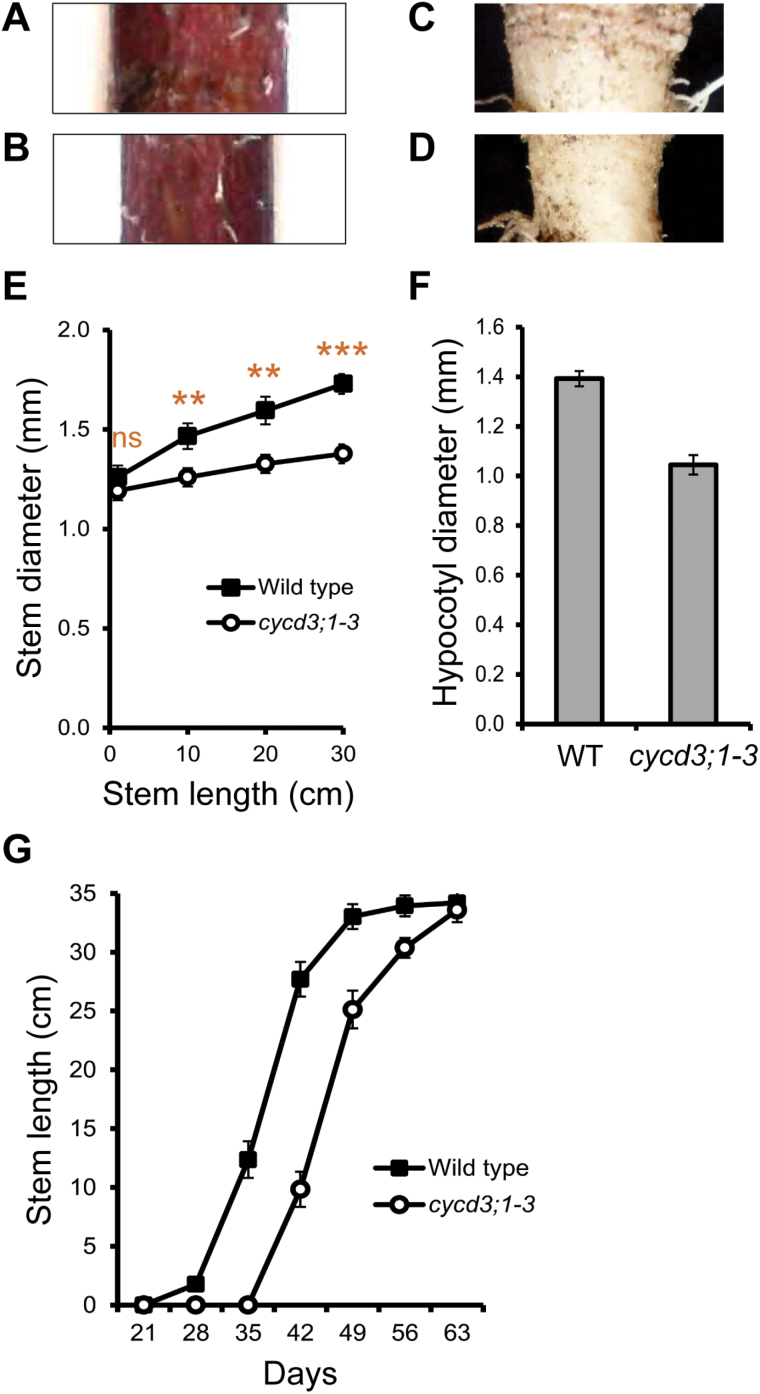
Loss of *CYCD3* causes retarded secondary growth and reduced organ size. (A, B) Representative examples of side views of the base of whole 30-cm-long inflorescences immediately above the rosette in wild-type (A) and *cycd3;1–3* (B) plants showing a thin-stemmed phenotype in *cycd3;1–3.* (C, D) Representative examples of side views of the base of whole mature hypocotyls from 30-cm-tall wild-type (C) and *cycd3;1–3* (D) plants showing a thin hypocotyl phenotype in *cycd3;1–3.* (E) Quantitative analysis of stem diameter at the base of 1, 10, 20, and 30-cm-long inflorescences of wild-type and *cycd3;1–3* plants showing the reduced growth in *cycd3;1–3.* Error bars represent standard error. Significance levels are shown as the difference between wild type and *cycd3;1–3* with *P* <0.01 and *P* <0.001 (*n=*10) indicated by double and triple asterisks, respectively (not statistically significant is indicated by ns). (F) Measurement of hypocotyl diameter in 30-cm-tall wild-type and *cycd3;1–3* plants showing the reduced growth in *cycd3;1–3.* Error bars represent standard error. Student’s *t* test showed that the difference between wild type and *cycd3;1–3* was extremely statistically significant (*P* <0.001; *n*=10). (G) Quantitative analysis of stem length in wild-type and *cycd3;1–3* plants at 21, 28, 35, 42, 49, 56, and 63 d (days relative to date of sowing) showing equivalent rates of stem elongation in wild type and *cycd3;1–3.* Student’s *t* test showed that wild type and *cycd3;1–3* were not significantly different in stem length at 63 d (*n*=10). Error bars represent standard error.

The highly overlapping patterns of expression observed for the three *CYCD3* genes during vascular development suggested that the CYCD3-directed CYCD/RBR/E2F pathway (Dewitte *et al.*, 2003; 2007) might play a specific role in regulating cambial cell proliferation. Therefore, the effect of reduced expression of CYCD3 on secondary growth was examined using a triple homozygous *cycd3;1 cycd3;2 cycd3;3* (*cycd3;1–3*) loss-of-function mutant (Dewitte *et al.*, 2007). Clear developmental defects were seen in *cycd3;1–3* plants. At the base of adult stems and mature hypocotyls from 30-cm-tall plants, corresponding to major zones of prominent secondary growth, *cycd3;1–3* was thinner compared with their wild-type equivalents (Fig. 3A–D). Radial growth at the stem base was monitored by measuring the diameter at different stages of stem elongation in wild-type and mutant plants. The *cycd3;1–3* mutant showed a substantially reduced radial growth rate with an approximately 20% reduction (*t* test *P* <0.001) in thickening growth compared with the wild type at the stage where wild-type inflorescences had reached 30cm in length (Fig. 3E). At the base of mature hypocotyls, mutants showed an approximately 25% reduction (*t* test *P* <0.001) in thickening growth (Fig. 3F). The reduced stem and hypocotyl phenotypes in *cycd3;1–3* closely resemble those reported in plants with mutations in known cambium regulators such as *WOX4* and *PXY/TDR* (Fisher and Turner, 2007; Hirakawa *et al.*, 2008, 2010; Ji *et al.*, 2010), and can be considered diagnostic of plants with reduced cambium activity.

Measurements of apical growth of the stem showed that, despite the emergence of the inflorescence being delayed in *cycd3;1–3* (see Supplementary Fig. S3 at *JXB* online), as previously reported (Dewitte *et al.*, 2007), the rate of stem elongation was comparable between wild type and mutant, with *cycd3;1–3* stems reaching the length of their wild-type equivalents toward the end of the growth period (Fig. 3G). These results show clearly that radial growth was more compromised than apical growth, suggesting that a decrease in the activity of the cambium was the major cause of the reduced thickening growth in the stem. Interestingly, delayed emergence and other perturbations in apical growth of the stem are characteristic features of *wox4* and *pxy* mutants (Fisher and Turner, 2007; Ji *et al.*, 2010). In summary, our data indicate an effect of loss of *CYCD3* expression on cambium activity, leading to retarded development in the stem and hypocotyl, and reduced organ size.

### CYCD3 promotes cambial cell proliferation in the inflorescence stem

**Fig. 4.**
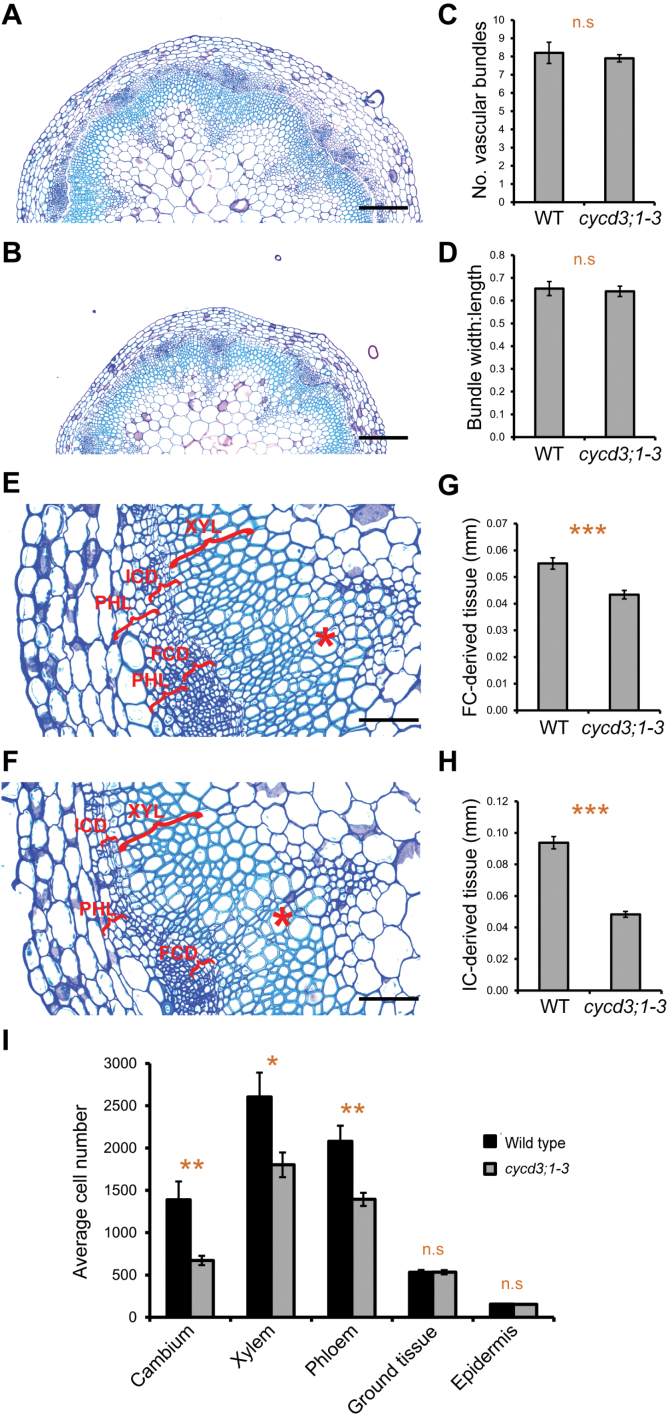
Loss of *CYCD3* leads to reduced cambial activity and vascular cell number in the inflorescence stem. (A, B) Histological representations at the base of 30-cm-long inflorescences immediately above the rosette showing tissue organization in wild-type (A) and *cycd3;1–3* (B) plants. (C) Measurement of vascular bundle number at the base of wild-type and *cycd3;1–3* stems. Error bars represent standard error. Student’s *t* test showed that wild type and *cycd3;1–3* were not significantly different (*n*=5). n.s, not statistically significant. (D) Measurement of the width:length (tangential:radial) ratio of vascular bundles at the base of wild-type and *cycd3;1–3* stems. Error bars represent standard error. Student’s *t* test showed that wild type and *cycd3;1–3* were not significantly different (*n*=15). n.s, not statistically significant. (E, F) Higher magnification histological representations of vascular tissue at the base of wild-type (E) and *cycd3;1–3* (F) stems. The lateral extension of the FC-derived (FCD) and IC-derived (ICD) tissue, and of the phloem (PHL) and xylem (XYL) tissues are indicated by red brackets. Asterisks indicate primary vascular bundles. (G, H) Measurement of lateral extension of the FCD (G) and ICD (H) tissues at the base of wild-type and *cycd3;1–3* stems. Error bars represent standard error. Student’s *t* tests showed that the differences between wild type and *cycd3;1–3* in both the FCD (G) and ICD (H) tissues were extremely statistically significant (*P* <0.001; *n*=15) indicated by triple asterisks. (I) Quantification of average cell number of different cell types in tissue sections at the base of wild-type and *cycd3;1–3* stems. Error bars represent standard error. Significance levels are shown as the difference between wild type and *cycd3;1–3* with *P* <0.05 and *P* <0.01 indicated by single and double asterisks, respectively (*n*=15). n.s, not statistically significant. Scale bars=200 μm (A, B), 100 μm (E, F).

To determine more precisely the effect of reduced CYCD3 activity on cambium-related cell division and differentiation processes, the vascular anatomy of the stem base of 30-cm-tall *cycd3;1–3* plants was analysed. At the base of adult stems, the vascular tissue is composed of radially oriented, closed cylinders of cambium (FC and IC) with an inner xylem and outer phloem which are, in turn, enclosed by a ground tissue (cortex and endodermis) and epidermis layers (Sehr *et al.*, 2010; Sanchez *et al.*, 2012). Different tissue layers are easily distinguished by the characteristic shapes and spatial division planes of the cells comprising each tissue. In terms of overall tissue patterning, the *cycd3;1–3* mutant was indistinguishable from the wild type with a normal arrangement of vascular and non-vascular tissue (Fig. 4A, B), and there was no significant difference in the average number of vascular bundles between wild-type and mutant stems (Fig. 4C). Furthermore, calculations of the width:length (tangential:radial) ratio of vascular bundles, a measure of vascular organization (Fisher and Turner, 2007), showed no significant difference between the wild type and *cycd3;1–3* (Fig. 4D). These results suggest that tissue patterning is not greatly affected upon loss of *cycd3;1–3*.

The FC and its immediate population of daughter cells have been defined as FC-derived (FCD) tissue, whereas the IC and its immediate population of daughter cells were defined as IC-derived (ICD) tissue and both can be used as a measure of cambium activity (Sehr *et al.*, 2010). The effect of loss of CYCD3 function on cambium activity in mature *cycd3;1–3* stems was analysed. Microscopic analysis of FCD and ICD in wild-type stems identified the small, uniform, flattened, and undifferentiated cells of the FC and IC organized into radial cell files (resulting from continuous periclinal cell divisions), surrounded by more rounded and less uniformly shaped, differentiating xylem, and phloem and associated parenchyma cells (not organized into radial cell files) (Fig. 4E). Observations in the *cycd3;1–3* mutant showed that the equivalent cell types were present and were phenotypically indistinguishable from the wild type (Fig. 4E, F), indicating that CYCD3 is not essential for the establishment and identity of the cambium. By contrast, quantification of the lateral extension of the cambium-derived tissue in the stem base showed *cycd3;1–3* to have an approximately 21% reduction (*t* test *P* <0.001) in FCD, and an approximately 48% reduction (*t* test *P* <0.001) in ICD, compared with the wild type (Fig. 4G, H), indicating that loss of CYCD3 causes a substantial decrease in the activity of the cambium.

The reduced cambium activity observed in *cycd3;1–3* suggested that both cambial cell number and the number of daughter cells in the xylem and phloem lineages could be affected. Quantification of cell numbers in the cambium, xylem, and phloem of *cycd3;1–3* and wild-type stems (see Supplementary Fig. S4 at *JXB* online; Fig. 4E, F) showed that the number of cells comprising the cambium in *cycd3;1*–*3* was reduced by approximately 52% (*t* test *P* <0.01) compared with the wild type, with the mutant also showing respective reductions in xylem and phloem cell number of approximately 31% (*t* test *P* <0.05) and 33% (*t* test *P* <0.01) (Fig. 4I), confirming that loss of CYCD3 causes a marked decrease in cambium activity, limiting cell number in the xylem and phloem cell lineages. Cell numbers were also quantified in the surrounding ground tissue (cortex plus endodermis) and epidermis (see Supplementary Fig. S4 at *JXB* online) tissue layers which are established during primary growth from the apical meristem. The ground tissue and epidermis layers were easily identified in cross-sections by their characteristic large cells, with a single cell layer of endodermis enclosed by a 4–5 cell thick cortex, in turn, enclosed by a single cell-layered epidermis (see Supplementary Fig. S4 at *JXB* online). No significant difference was found in the number of cells comprising both the ground tissue and epidermis between the wild type and *cycd3;1–3* (Fig. 4I), supporting the proposition that decreased cambium activity, as opposed to a defective apical meristem, was the major cause of the reduced vascular cell number and overall radial organ size in *cycd3;1–3* stems.

**Fig. 5.**
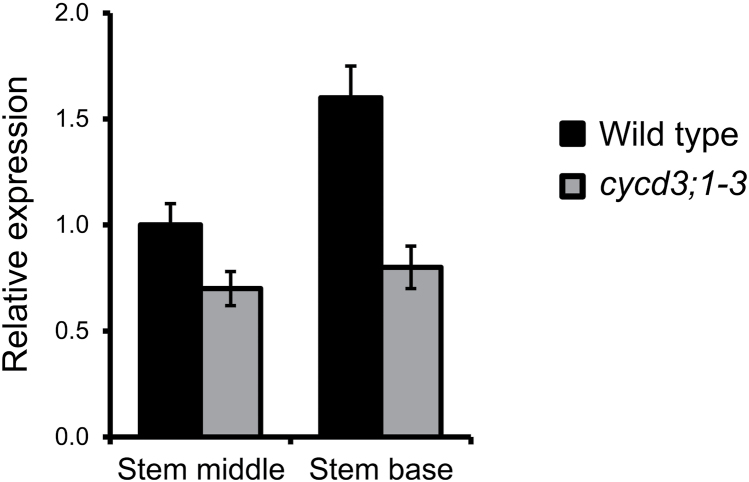
Expression analysis of the *CYCB1;1* mitotic marker gene in the inflorescence stem. Reverse transcription quantitative PCR was used to assay *CYCB1;1* transcript abundance in the middle and base of 30-cm-long stems of wild-type and *cycd3;1–3* plants using *ACTIN2* as the endogenous reference gene. Relative transcript abundance is scaled to expression in the middle stem of the wild type. Error bars represent standard error.

Importantly, the vascular cell populations which exhibited the phenotypic effects upon loss of *CYCD3* overlapped the domains in which *CYCD3* genes were shown to be expressed (Fig. 2). To assess the status of mitotic cell cycle activity in developing vascular tissue in *cycd3;1–3* stems, reverse transcription quantitative PCR (RT-qPCR) was performed to monitor the expression of the mitotic B-type cyclin gene *CYCB1;1*, a commonly-used marker of mitotic cell cycle activity during plant development (Coloń-Carmona *et al.*, 1999). Consistent with reduced cambial cell division in *cycd3;1–3* stems, mutants showed reduced *CYCB1;1* expression compared with the wild type in the middle and base of stems, an effect that was more pronounced in the stem base (Fig. 5), confirming that reduced cambial cell number in *cycd3;1–3* stems is associated with reduced mitotic cell cycle activity. Taken together, our results demonstrate that CYCD3 plays a significant positive role in the regulation of cambium activity in the stem and that its loss leads to retarded secondary growth and reduced organ size.

### CYCD3 restrains cell expansion and differentiation of cambial cells into xylem cells

**Fig. 6.**
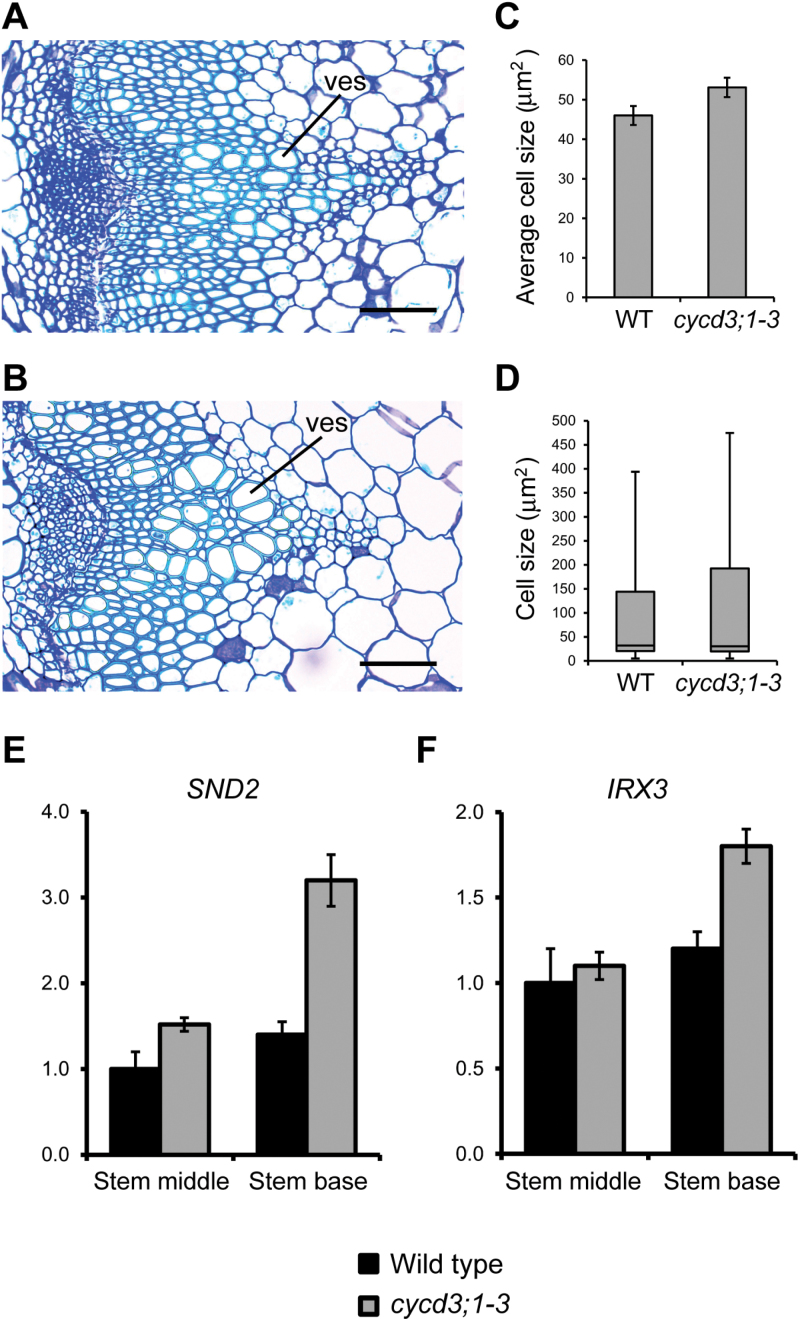
Loss of *CYCD3* leads to increased xylem cell size and differentiation in the inflorescence stem. (A, B) High magnification histological representations at the base of 30-cm-long inflorescences showing xylem cells in primary and secondary vascular tissue in wild-type (A) and *cycd3;1–3* (B) plants. (C) Measurement of xylem cell size at the base of 30-cm-tall wild-type and *cycd3;1–3* plants showing the increased average cell size in *cycd3;1–3.* Error bars represent standard error. Student’s *t* test showed that the difference between the wild type and *cycd3;1–3* was statistically significant (*P* <0.05; *n* >2000). (D) Box plot representation of xylem cell size at the base of 30-cm-tall plants showing the distribution of xylem cell sizes in wild-type and *cycd3;1–3* stems. Displayed are the maximum and minimum values, median, and 30th and 95th percentiles. Error bars represent standard error. (E, F) Expression analysis of the xylem cell differentiation marker genes *SND2* (E) and *IRX3* (F) in the middle and base of 30-cm-long stems of wild-type and *cycd3;1–3* plants by RT-qPCR. Transcript abundance was normalized using *ACTIN2* as the endogenous reference gene. Relative transcript abundance is scaled to expression in the middle stem of the wild type. Error bars represent standard error. ves, xylem vessels. Scale bars=100 μm (A, B).

An additional phenotype that was noted at the stem base of *cycd3;1–3* mutants was the presence of enlarged xylem cells, particularly those of vessels (Fig. 6A, B). *Arabidopsis CYCD3* genes have been shown previously to mediate the balance between cell division and cell expansion during leaf development by regulating the duration of the ‘mitotic window’ and the onset of cell expansion and associated endoreduplication events (Dewitte *et al.*, 2007). To determine the overall effects of loss of CYCD3 function on cell expansion in the xylem cell lineage, xylem cell sizes in the stem base of 30-cm-tall *cycd3;1–3* and wild-type plants was quantified. Overall, it was found that average xylem cell size in *cycd3;1*–*3* was increased by approximately 13% (*t* test *P* <0.05) compared with the wild type (Fig. 6C). Consistent with an overall shift to increased xylem cell size, the mutant showed a greater range of cell sizes with a significant number of cells reaching sizes not seen in the wild type (Fig. 6D). These results indicate that loss of CYCD3 causes a significant enhancement in cell growth in developing xylem cells. The apparent enlargement of xylem cells observed in *cycd3;1–3* stems suggested that the cellular transition from division to differentiation pathways in the xylem could be affected. To evaluate further the effects of loss of CYCD3 on the progression of vascular differentiation in stems, RT-qPCR was used to determine the expression of two well-characterized markers of xylem cell differentiation, *SECONDARY WALL-ASSOCIATED NAC DOMAIN PROTEIN2* (*SND2*) and *IRREGULAR XYLEM3* (*IRX3*) which, respectively, encode a transcription factor and a cellulose synthase gene that are tightly associated with xylem secondary cell wall biosynthesis (Brown *et al.*, 2005; Zhong *et al.*, 2008). Consistent with an overall shift toward differentiation in the developing xylem of *cycd3;1–3* stems, mutants showed an up-regulation of *SND2* and *IRX3* expression compared with the wild type of approximately 2.3-fold (130%) and 1.5-fold (50%), respectively, an effect that was more pronounced in the stem base (Fig. 6E, F).

To exclude the possibility that the increased *IRX3* and *SND2* expression observed in the mutant was the result of an increase in the proportion of xylem cells (relative to the total of all other cell types) in the stem, the proportional contribution of xylem cells was calculated using data derived from stem cross-sections presented in Fig. 4I. The wild type had an average xylem cell number of 2605 and an average total number of all cell types of 6757.2, whereas the mutant had respective numbers of 1801.6 and 4553.2. Calculation of the proportion of xylem cells in stems showed both lines to be almost identical, with the wild type having 39% xylem (2605/6757.2) and the mutant having 40% xylem (1801.6/4553.2), ruling out an increase in the proportion of xylem cells as the cause of increased *SND2* and *IRX3* expression.

Importantly, the *CYCD3* genes were shown to be specifically expressed in the cambium during stem vascular development and absent from older xylem cells (Fig. 2M, N), suggesting that a key point of regulation of cell expansion/differentiation processes by CYCD3 may occur in xylem cell precursors and their immediate progeny cells. Together, our data suggest that CYCD3 plays an important additional role in stem vascular development, in restraining cell expansion and differentiation in developing xylem cells.

## Discussion

In contrast to our relatively good understanding of the regulation of primary meristems, remarkably little is known concerning the molecular mechanisms controlling cell division in the cambial meristem and its integration with vascular differentiation. In this work, the wider role of the cell cycle in vascular development was investigated and the *Arabidopsis CYCD3* subgroup of cell-cycle genes were identified as being key regulators of cambial cell proliferation and secondary growth. The *CYCD3* genes were shown previously to control the balance between cell division and cell expansion during leaf and petal development (Dewitte *et al.*, 2007), and CYCD3 is also required for normal cell division and developmental rate during embryogenesis (Collins *et al.*, 2012). In a mutant lacking all three *CYCD3* genes (*cycd3;1–3*), it was found that radial organ size was severely affected with mutant stems and hypocotyls showing a marked reduction in diameter linked to reduced cell division in the cambium, leading to decreased xylem and phloem cell numbers. Conversely, it was found that the reduced xylem cell number was accompanied by an increase in xylem cell size and differentiation. Interestingly, perturbation of *CYCD3* levels in transgenic plants through the constitutive over-expression of *CYCD3;1* was shown to retard secondary xylem formation resulting from an inhibition of cell differentiation associated with reduced radial growth (Dewitte *et al.*, 2003). This apparent reciprocal phenotype to the CYCD3 triple loss-of-function mutant phenotype analysed in this study, which exhibits enhanced xylem cell differentiation, indicates that correct levels of CYCD3 activity is required for normal xylem development and radial organ growth. Importantly, all three CYCD3 genes showed activity in the procambium and cambium, linking CYCD3 expression to the stem cell populations that drive both primary and secondary vascular development. Our data show that developmental stage- and cell-type-specific regulation of CYCD3 is required to promote mitotic cell-cycle activity in the cambium and that its down-regulation is required for the progression of cell expansion and differentiation of vascular stem cells into xylem cells, providing the first conclusive evidence of a direct link between the cell cycle and vascular development in controlling plant organ growth.

The vascular development phenotypes observed in *cycd3;1–3* closely resemble those reported in plants where the expression of known cambium regulators were manipulated, including *wox4* and *pxy/tdr* (Fisher and Turner, 2007; Hirakawa *et al.*, 2008, 2010; Ji *et al.*, 2010; Suer *et al.*, 2011), *hca2* (Guo *et al.*, 2009), *rul1* (Agusti *et al.*, 2011), or *wox14* (Etchells *et al.*, 2013). In these mutants, insufficient cambial cell divisions contributed to the inhibited thickening growth in different organs. Significantly, no effects on the orientation of cell division or overall vascular organization were observed in the *cycd3;1–3* mutant, indicating a distinct role for CYCD3 in processes that specifically control the rate of cambial cell division. In this respect, the *cycd3;1–3* mutant phenotype most closely resembles those seen in mutants of *WOX4* and *WOX14*, which have already been shown to regulate stem cell proliferation independently of processes that control vascular organization (Etchells *et al.*, 2013).

A number of developmental, hormonal, and environmental signals are known to regulate procambium/cambium activity (Miyashima *et al.*, 2013), which presumably must ultimately impinge on cell-cycle regulation. The phytohormones auxin and cytokinin play particularly important roles during both primary and secondary growth by controlling cambial cell proliferation (Little *et al.*, 2002; Scarpella *et al.*, 2006; Matsumoto-Kitano *et al.*, 2008). Several lines of experimental evidence support a role for CYCD3 in the integration of multiple input signals to control procambium/cambium activity. *CYCD3;1* responds positively to auxin and cytokinin in line with its proposed role as a regulator of the G1/S phase transition in response to external signals (Riou-Khamlichi *et al.*, 1999; Oakenfull *et al.*, 2002; Dewitte *et al.*, 2007). Consistent with a role downstream of hormone signalling, the expression patterns revealed for *CYCD3s* in the procambium and cambium of the stem (Fig. 2) overlap domains of activity of the DR5 reporter, a commonly used marker of localized auxin accumulation (Ibañes *et al.*, 2009). Recent work has shown *CYCD3;3* as a direct target of DNA-binding with one finger 3.4/OBF-binding protein 1 (DOF3.4/OBP1), a member of the DOF group of plant-specific transcription factors (DOF TFs) proposed to have central roles in vascular development (Skirycz *et al.*, 2008; Le Hir and Bellini, 2013). DOF3.4/OBP1 was found directly to bind and regulate the *CYCD3;3* promoter *in vitro* and, like all three *CYCD3s*, was shown to be expressed in the procambium of the stem (Skirycz *et al.*, 2008). Another DOF TF, DOF5.6/HCA2, was shown to promote cell proliferation in the interfascicular cambium (Guo *et al.*, 2009). Intriguingly, a number of DOF TFs including DOF3.4/OBP1 are known to be regulated by auxin (Kang and Singh, 2000). Thus, CYCD3 could provide a link between hormone signalling and cell-cycle activation in cambial cells through distinct DOF TFs and *CYCD3* genes.

The additional phenotype observed in the *cycd3;1–3* mutant of increased xylem cell expansion was associated with the up-regulation of xylem secondary cell wall biosynthetic genes, suggesting that CYCD3 is required to restrain cell expansion/differentiation in the xylem cell lineage. As expression of CYCD3 was observed specifically in the cambium during vascular development (Fig. 2), the most likely scenario is that CYCD3 exerts control over cell expansion/differentiation in xylem cell precursors and their immediate progeny cells and that subsequent CYCD3 down-regulation in cells displaced from the cambium is required for continued cell expansion and differentiation in xylem cells. This is supported by observations of xylem development in plants over-expressing *CYCD3;1*, where elevated *CYCD3;1* levels lead to inhibited differentiation and retarded xylem formation (Dewitte *et al.*, 2003). The proposed role for CYCD3 in restraining cell expansion/differentiation of vascular cells appears to parallel its role in restraining cell expansion/differentiation during leaf and petal development (Dewitte *et al.*, 2007). In leaves, CYCD3 acts to inhibit endocycles, and its loss results in unrestrained endocycles, associated with enhanced cell expansion and differentiation (Dewitte *et al.*, 2007). It is notable that xylem cells in different plant species are known to have both endocycles and substantial cell growth, to enable them to perform their specialized functions (Martίnez-Pérez *et al.*, 2001; Prieto *et al.*, 2004). Further work will be required to elucidate the precise mode of action of CYCD3 in restraining cell expansion and differentiation in the xylem cell lineage, and whether CYCD3 interacts with the endocycle in this regulation, as it does in leaves (Dewitte *et al.*, 2007).

It is concluded that CYCD3 is an important regulator of cambial cell division, determining the number of proliferating cells and their progeny in the xylem and phloem, and the size of xylem cells during vascular development. Tight control of the balance between cell division and cell differentiation/expansion mediated by CYCD3 is critical for proper vascular development and secondary growth, which ultimately determines radial organ size.

## Supplementary data

Supplementary data can be found at *JXB* online.

Supplementary Fig. S1. Confirmation of the genotype of the *cycd3;1–3* line by RT-PCR.

Supplementary Fig. S2. Radial distribution of expression levels of 96 core cell-cycle genes and four cambium marker genes based on expression profile data across 19 different cell and tissue types of the root tip (Brady *et al.*, 2007).

Supplementary Fig. S3. Illustration of delayed flowering in the *cycd3;1–3* line compared with the wild type.

**Supplementary Fig. S4**. Example of cell counting using transverse sections at the base of 30-cm-long inflorescences immediately above the rosette.

Supplementary Data
