# Supplementary material for: CYCD3 D-type cyclins regulate cambial cell proliferation and secondary growth in Arabidopsis
Source: J Exp Bot. 2015 May 28;66(15):4595–606. doi: 10.1093/jxb/erv218 (PMC4507761; doi:10.1093/jxb/erv218)
Supplement: Supplementary Data [file supp_66_15_4595__index.html]

CYCD3 D-type cyclins regulate cambial cell proliferation and secondary growth in Arabidopsis — CYCD3 D-type cyclins regulate cambial cell proliferation and secondary growth in Arabidopsis — Supplementary Data 

# CYCD3 D-type cyclins regulate cambial cell proliferation and secondary growth in *Arabidopsis*

## Supplementary Data

Data files

**Files in this Data Supplement:**

- Supplementary Data - Supplementary Data
