# Supplementary material for: CYCD3 D-type cyclins regulate cambial cell proliferation and secondary growth in Arabidopsis
Source: J Exp Bot. 2015 May 28;66(15):4595–606. doi: 10.1093/jxb/erv218 (PMC4507761; doi:10.1093/jxb/erv218)

CYCD3 D-type cyclins regulate cambial cell proliferation and secondary growth in *Arabidopsis.*

Carl Collins, Maruthi M.N and Courtney E. Jahn

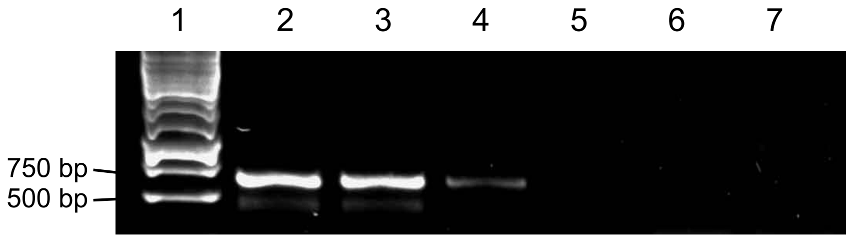

CYCD3 D-type cyclins regulate cambial cell proliferation and secondary growth in *Arabidopsis.* Carl Collins, Maruthi M.N., Courtney E. Jahn.

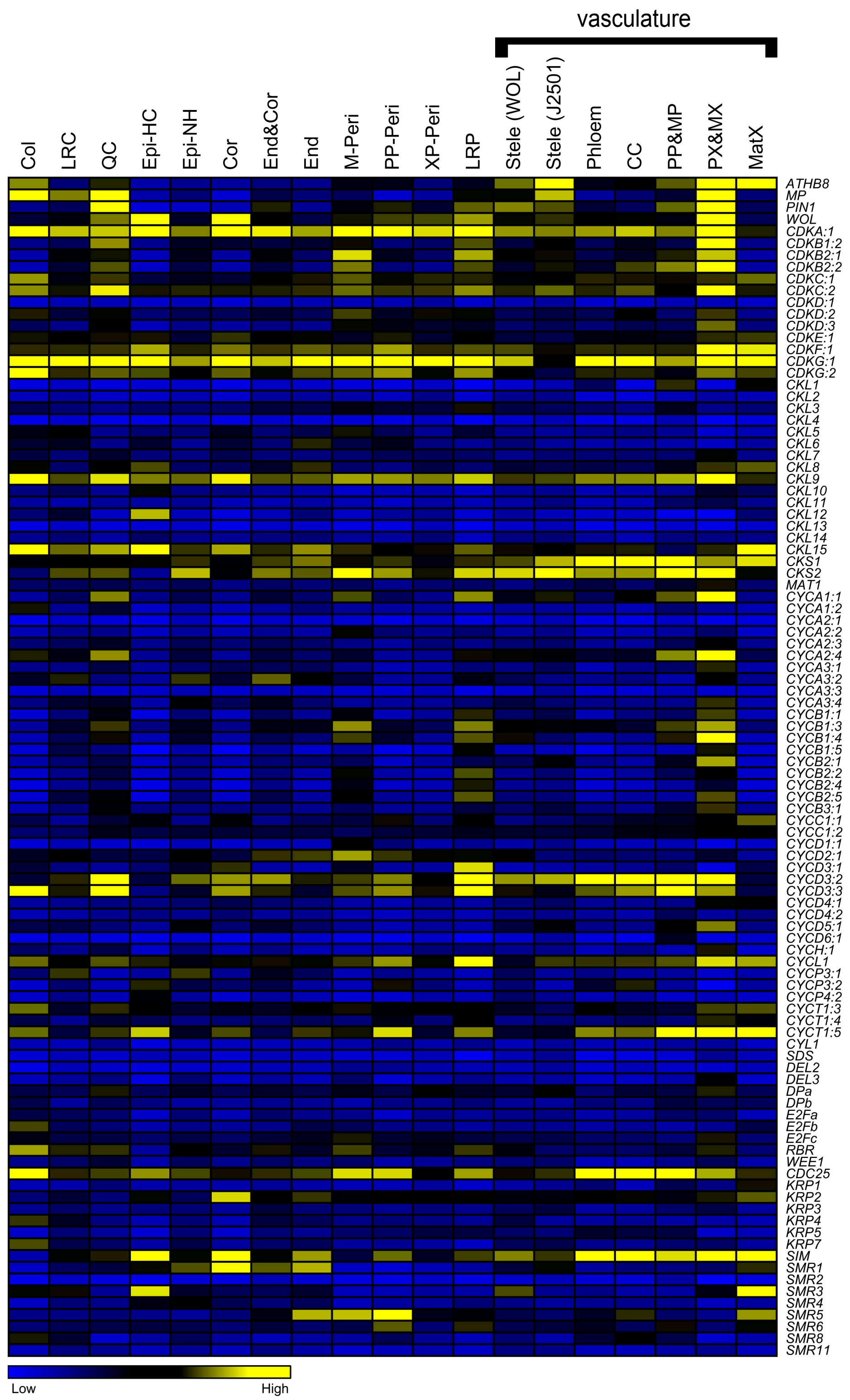

CYCD3 D-type cyclins regulate cambial cell proliferation and secondary growth in *Arabidopsis.*

Carl Collins, Maruthi M.N and Courtney E. Jahn

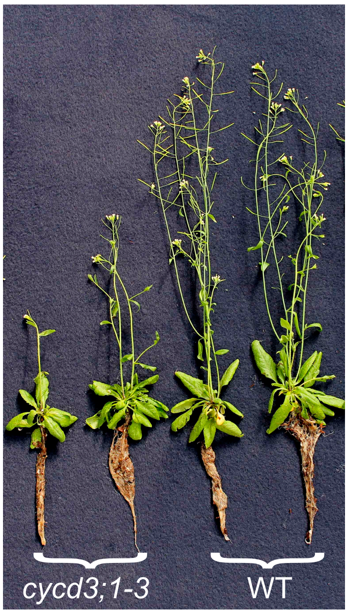

CYCD3 D-type cyclins regulate cambial cell proliferation and secondary growth in *Arabidopsis.*

Carl Collins, Maruthi M.N and Courtney E. Jahn

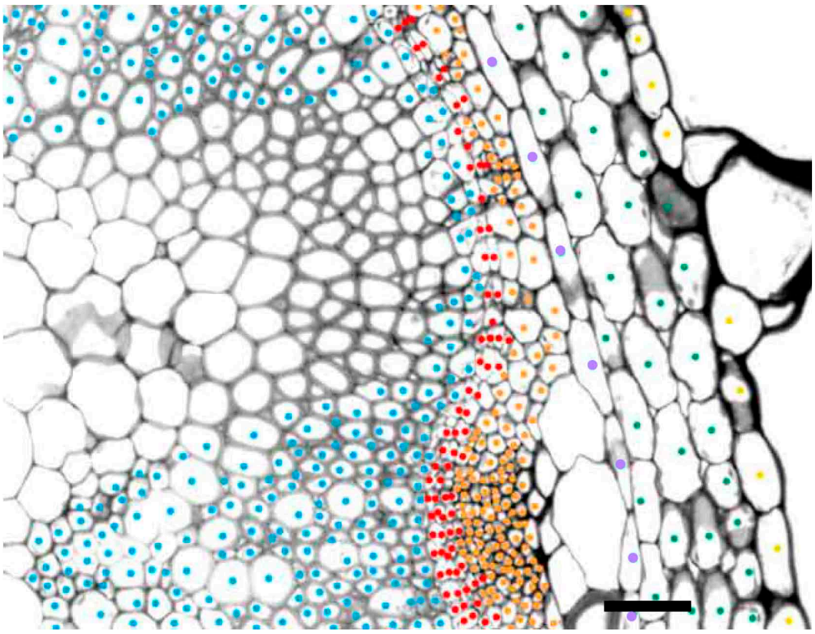

Supplement: Supplementary Data [file supp_erv218_jexbot137620_file001.pdf]
